# Development of an electronic navigation system for elimination of examiner-dependent factors in the ultrasound screening for developmental dysplasia of the hip in newborns

**DOI:** 10.1038/s41598-020-73536-9

**Published:** 2020-10-02

**Authors:** Alexander Kolb, Catharina Chiari, Markus Schreiner, Stephan Heisinger, Madeleine Willegger, Gregor Rettl, Reinhard Windhager

**Affiliations:** Department of Orthopedics and Trauma-Surgery, Medical University of Vienna, Vienna, Austria

**Keywords:** Paediatric research, Bone imaging, Ultrasonography, Anatomy

## Abstract

To develop an electronic navigation system to increase reliability and comparability in the ultrasound screening of developmental dysplasia of the hip (DDH). The impact of the navigation system on transducer positioning and on sonographic measurements according to Graf was analyzed. Twenty hips in newborns were examined sonographically using a new navigation system capable of detecting the transducer and pelvis position in order to calculate the relative tilt in the frontal, axial, and sagittal-plane. In each newborn an ultrasound image was obtained conventionally according to Graf and a second image using the sonographic navigation system. Relative roll and pitch angles and sonographic measurements were analyzed using paired T-tests and Levene-tests. Relative tilt angles in the conventional group ranged from − 8.9° to 14.3° (frontal-plane) and − 23.8° to 14.2° (axial-plane). In the navigation-assisted group ranges from − 3.0° to 3.5° and − 2.8° to 4.5° were observed. Variances were significantly lower in the navigation-assisted group (*p* < 0.001 and *p* = 0.004 respectively). The navigation system allowed for a significant reduction of relative tilt angles between the transducer and the newborn pelvis, thus supporting an optimal transducer positioning. This is a promising approach to improve reproducibility and reliability in the ultrasound screening for DDH.

## Introduction

The ultrasound screening according to Graf is broadly accepted as the gold standard for the early detection of developmental dysplasia of the hip (DDH) in newborns^1,2^. This method is based on a high degree of standardization of the sonographic examination to ensure reproducibility of the results^3,4^. Despite these efforts, examiner-related factors have been discussed to potentially influence incidence rates of DDH measured sonographically^5^. Thus, examiner-related factors were addressed using assistive devices such as a mechanical transducer guiding device, a positioning aid for the baby (cradle) and most importantly well-structured training of the examiners^6,7^. In regard to examiner-related errors the relative tilt between the examined hip and the ultrasound transducer is a key factor, which has been shown by Jaremko et al. using 3D ultrasound imaging^8^. Moreover, the effect of relative tilt angles between hip joint and transducer has also been analyzed using Graf’s method, which is as a gold standard performed routinely by 2D ultrasound imaging, based on an optoelectronic motion capture system^9^. Here the effect of the relative tilt angles on sonographic measurements and furthermore on the DDH classification according to Graf proved to be highly significant^9^. However, the reported optoelectronic motion capture system setup was not capable of determining the position of the newborn’s hip or pelvis, which is a major disadvantage limiting further analyses in routine use.

The aim of this study was to develop a new electronic navigation system to minimize examiner-related errors in regard to relative tilt angles between the ultrasound transducer and the examined hip joint and to analyze effects of such a system on relative tilt errors in comparison to the conventional ultrasound screening according to Graf.

## Materials and methods

The study was approved by the institutional review board of the Medical University of Vienna. All methods were carried out in accordance with relevant guidelines and regulations. All legal guardians gave their informed consent prior to the inclusion in this study.

**Figure 1:**
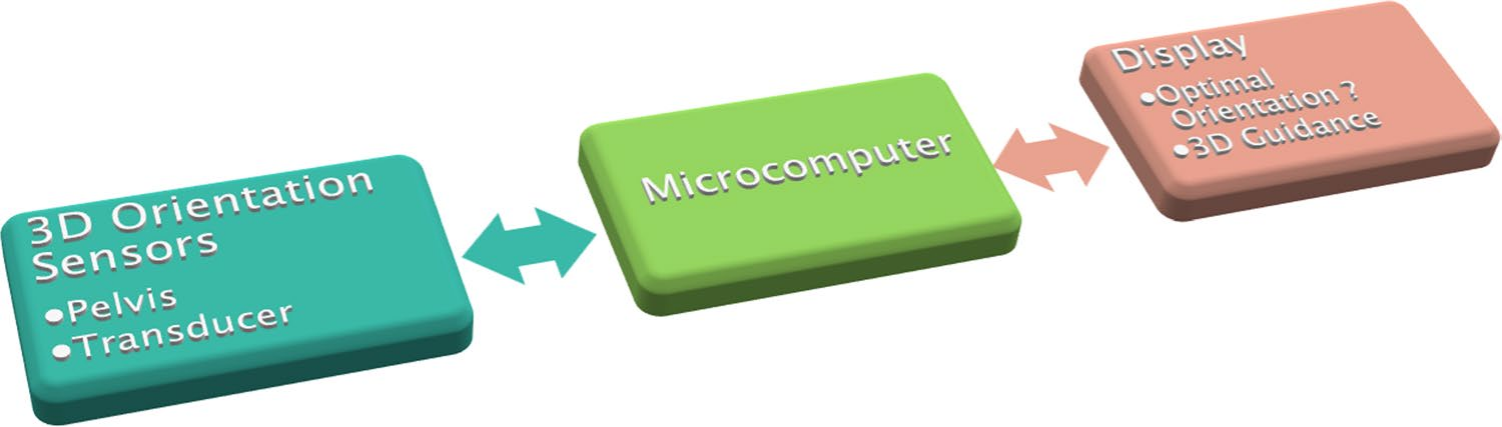
Systematic setup of the electronic navigation system: The 3D-orientation-sensors communicate the data to a microcomputer, which further processes the data and transfers them to a PC-based output software, which stores the data and depicts the relative angles to assist the navigation.

**Figure 2:**
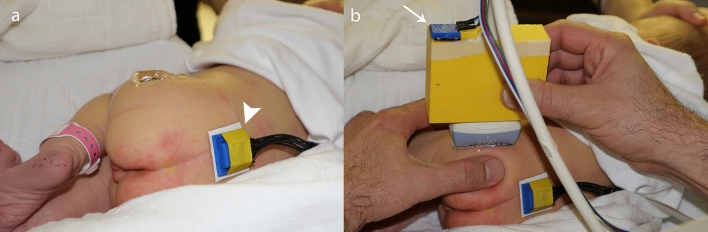
Illustration of the positioning of 3D-orientation-sensors: (**a**) epicutaneous fixation of the pelvic sensor (arrow head) dorsal above the sacral bone to detect the pelvis position, (**b**) fixation of the second sensor (thin arrow) to the transducer using a 3D printed adapter in a typical situation prior to adjustment of the transducer position.

**Figure 3:**
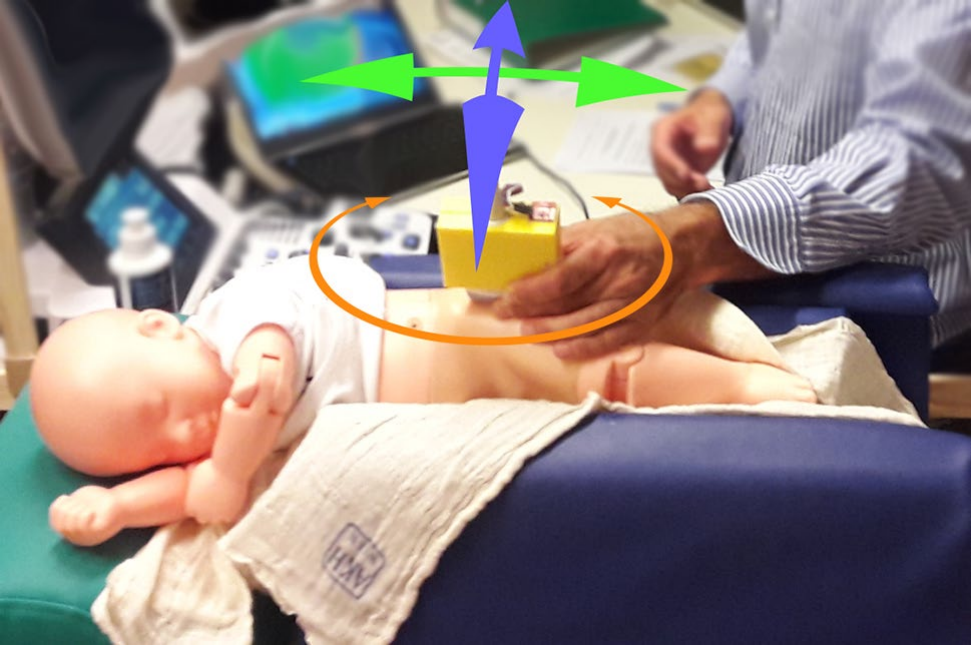
Illustration of tilt and rotational movements: roll-angle in the frontal plane (green double arrow), pitch-angle in the axial plane (blue double arrow), and yaw-angle in the sagittal plane (orange double arrow).

A new electronic navigation system was developed at our institution in order to detect potential examiner-related influences and errors (see Fig. 1). The microprocessor-based system consists of two position sensors, one of which is attached to the ultrasound transducer using a 3D printed adapter whereas the other one is attached epicutaneously in a median position dorsal to the sacrum (see Fig. 2). Both position sensors are composed of an accelerometer, a gyroscope and a magnetometer (MPU-9250, InvenSense Inc., San Jose, CA, USA). A specifically designed software program processes the measurements and calculates the relative tilt between both sensors in the frontal, axial and sagittal plane respectively representing the roll, pitch and yaw angle (see Fig. 3). The afore-mentioned measurements are hence communicated to a PC-based output software, which stores the data and depicts the angles to assist the navigation. The measurement accuracy of the roll and pitch angle was determined to be ± 1° and for the yaw angle it was determined to be ± 2°. A patent application for this system has been filed^10^.

Ten consecutive newborns (20 hips) were included into the study and screened sonographically by a single experienced examiner according to Graf’s method within the first neonatal week. Ultrasound screening was performed using a GE Logiq F8 series system and a 7.5-MHz LH linear transducer (GE Healthcare, Milwaukee, WI, USA). The examination was performed in lateral position using a special positioning device (Sono-Fix, Gebrüder Hirschbeck GmbH, Austria) as recommended by Graf^7^. A feed-and-sleep technique was used to reduce spontaneous movements of the newborns during the measurement. For every examined hip joint two ultrasound images were obtained. First, an ultrasound image was obtained according to the sonographic criteria described by Graf^6^ (conventional group). Second, an additional ultrasound image was obtained using the navigation system in addition to Graf’s sonographic criteria to optimize the transducer position according to the displayed tilt angles (navigation-assisted group). If no representative image could be obtained due to agitation of the newborn the collection of data was aborted.

### Statistical analysis

Data were processed using the SPSS Statistics 26 software (SPSS Inc., Chicago, IL, USA). Normal distribution of parameters was evaluated using Kolmogorov–Smirnov tests. Relative roll, pitch and yaw angles between transducer and pelvic were analysed using paired t-tests and Levene-tests. Because of the coarsening nature of classification systems sonographic α- and β-angels were analyzed directly using paired t-tests^11^. The level of significance was defined as *p* < 0.05.

## Results

The feed-and-sleep technique used to reduce spontaneous movements was sufficient in all examined newborns. In seven of the ten newborns both hips were measured according to the study protocol, in three newborns only one hip was measured due to increasing spontaneous movements during the examination. Thus, in total data from 17 hips (34 ultrasound images) were obtained according to the study protocol.

**Figure 4:**
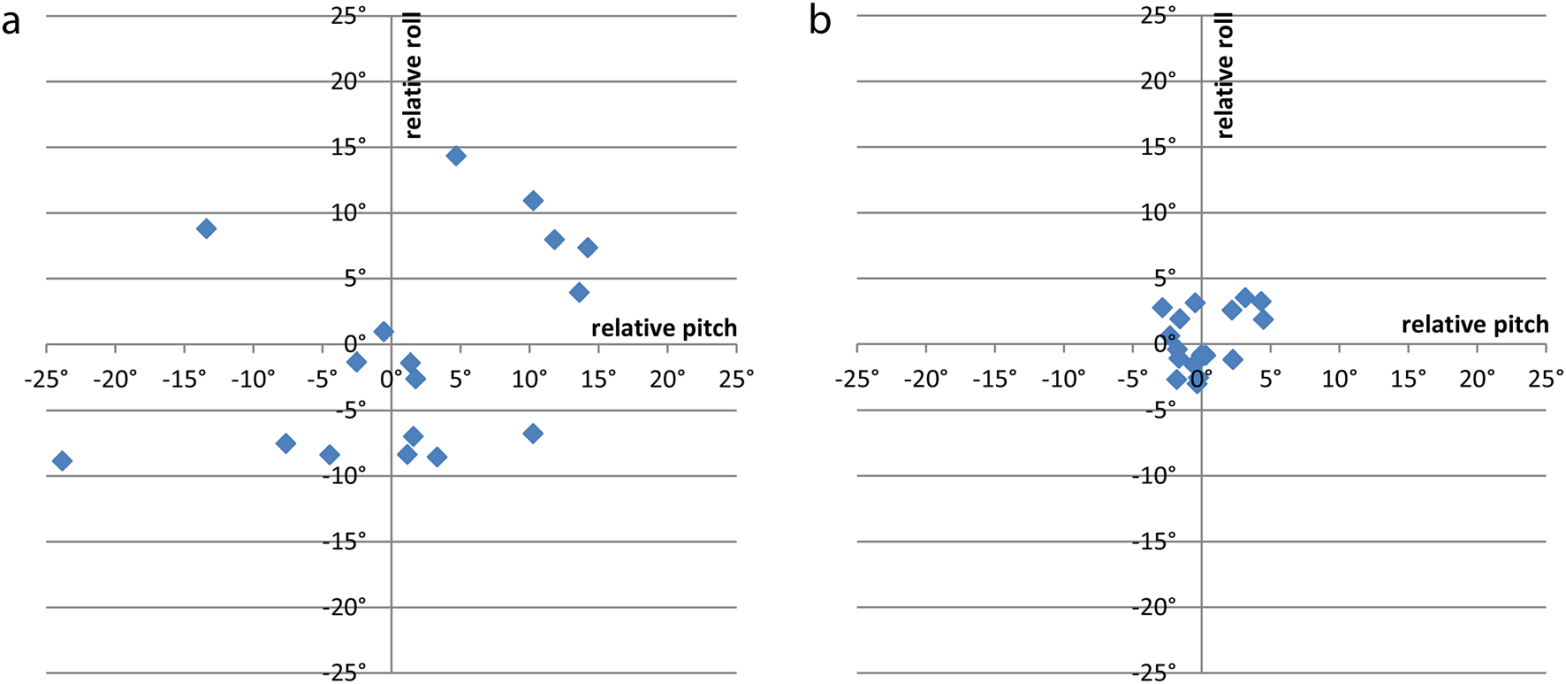
XY-plot of relative roll and pitch angles: (**a**) conventional group with adjustment of the transducer according to Graf’s sonographic criteria, (**b**) navigation-assisted group using the navigation system in addition to Graf’s criteria to optimize the transducer position.

In the conventional group, where sonographic images were obtained without navigation-assistance, relative tilt angles between transducer and pelvis ranged from − 8.9° to 14.3° (roll angle measured in the frontal plane) and from − 23.8° to 14.2° (pitch angle measured in the axial plane). In the navigation-assisted group relative tilt angles ranged from − 3.0° to 3.5° (roll angle in the frontal plane) and − 2.8° to 4.5° (pitch angle measured in the axial plane) respectively (see Table 1). Levene’s test of variance showed significant differences in the relative tilt angles in the frontal and axial plane with lower variance in the navigation-assisted study group (roll angle, *p* < 0.001; pitch angle, *p* = 0.004). The testing for mean values in the frontal and axial plane yielded no significant differences (roll angle, *p* = 0.678; pitch angle, *p* = 0.666). A visualization of the measured relative roll and pitch angles in both groups is given in Fig. 4.

**Table 1:** Overview of relative roll and pitch angles (tilt of transducer and pelvis in frontal and axial planes) conventional and navigation assisted.

	n	Minimum (°)	Maximum (°)	Mean (°)	SD
Roll angle (conventional)	17	− 8.9	14.3	− 0.4	7.96
Pitch angle (conventional)	17	− 23.8	14.2	1.3	9.58
Roll angle (navigation-assisted)	17	− 3.0	3.5	− 0.3	2.23
Pitch angle (navigation-assisted)	17	− 2.8	4.5	− 0.2	2.33

The afore-mentioned yaw angle (measured in the sagittal plane) could not be used for navigation due to the lack of validated basis data for the definition of a target value, thus no testing between conventional and navigation-assisted group was performed for this parameter. The mean value for the yaw angle showed a rotation of the ultrasound transducer versus the sacral sensor plane of 25.1° to the front (ranged from 6.8° to 43.1°).

Sonographic results according to Graf’s grading system were type I in 14 (82%) and IIa in 3 (18%) hips. Mean alpha angles were 62.6° (range 57–68°) in the conventional group and 61.6° (range 55–67°) in the navigation-assisted group; mean beta angles were 65.5 (range 55–73°) and 63.3° (range 48–75°). Differences of sonographically measured alpha and beta angles between the two groups were not significant (*p* = 0.255 and *p* = 0.312).

## Discussion

Several recent reports have highlighted the importance of correct ultrasound transducer positioning in relation to the pelvis to achieve reliable results in the ultrasound screening for DDH^8,9,12^. Furthermore, a susceptibility of Graf’s method to relative tilt errors was discussed intensively^9,13^. Despite the fact that reproducibility and reliability of Graf’s method was proved to be sufficient for screening purposes when repeated measurements on given ultrasound images are analyzed^14^, the importance of an optimal transducer positioning in the ultrasound examination prior to this step was reported. In an experimental study it has been shown that Graf’s sonographic criteria allow notable inclinations of the transducer position, which leads to significant alterations of the sonographic measurements^9^. These findings were supported by reports based on 3D ultrasound confirming the importance of an optimal positioning of the transducer plane^8,12^. However, these previous reports have focused on an optimal transducer position (i.e. measurement plane) without capturing the pelvis position. This represents a major limitation considering the importance of relative tilt angels between transducer and pelvis (i.e. hip joint). Based on this consideration, the presented electronic navigation system was developed, which is capable to detect both, the pelvis and the transducer position, in order to overcome this limitation.

As a main result this study demonstrates that the variance of relative tilt angles between the pelvis and the transducer can be reduced significantly using the electronic navigation system (see Fig. 4): In detail, our results showed this effect for both navigated angles i.e. the roll angle measured in the frontal plane and pitch angle measured in the axial plane (see Fig. 3).

In contrast, considering the mean values of the relative tilt angles, there was no significant difference between the conventional and the navigation-assisted study group. Therefore it can be concluded that the navigation system enables a more accurate alignment of the ultrasound transducer to the pelvis (reduced variance), while the principle of alignment remains unchanged compared to the sonographic method according to Graf (mean tilt angles in the frontal and axial plane around 0°). As a consequence, the electronic navigation system seems promising for minimizing the majority of relative tilt errors. However, it has to be considered that this finding is based on one experienced examiner; further studies are required to test the effect on mean angles for examiners of different experience levels.

In comparison to mechanical devices supporting the transducer in order to avoid transducer tilts as recommended by Graf (“Sono Guide”) the first evident advantage of the electronic navigation system is the detection of the pelvis position. From our experience, the spontaneous pelvis position of newborns in lateral position tends to be tilted frequently even using a cradle-like positioning aid for the baby as suggested by Graf. Such tilted pelvic positions are not detected in the conventional set-up, leaving the correction of such misalignments to the experience of the examiner. A further advantage of the electronic navigation system is the possibility to use the 3D-position-data for documentation of a correct examination process.

Besides the potential practical applicability of the electronic navigation system in the ultrasound screening for DDH, the detection of 3D-position-data are of further interest in this research field. While the alignment in the frontal and axial plane (roll and pitch angle) is defined to be parallel (target value of 0° between transducer and pelvic sensor in these planes), there is no defined target value for the sagittal plane (yaw angle) representing the angle between the optimal transducer position and the sensor position epicutaneous to the dorsal sacrum plane. Thus the relative yaw angle could not be used for navigation in this study. However, the device is capable of determining the yaw angle as shown, which might be used in further studies to optimize the transducer position also in the third plane.

## Limitations

Due to the cohort size the effect of the presented navigation system on ultrasound measurements could not be definitely assessed.

## Conclusion

The presented electronic navigation system is a novel approach to further improve the methodological reliability and quality of ultrasound screening for DDH. As the main result the system proves to be efficient to significantly reduce tilt errors. Further studies are required to prove the clinic impact of this promising approach.
